# Frequency‐Selective, Multi‐Channel, Self‐Powered Artificial Basilar Membrane Sensor with a Spiral Shape and 24 Critical Bands Inspired by the Human Cochlea

**DOI:** 10.1002/advs.202400955

**Published:** 2024-06-17

**Authors:** Eun‐Seok Jeon, Useung Lee, Seongho Yoon, Shin Hur, Hongsoo Choi, Chang‐Soo Han

**Affiliations:** ^1^ Department of Mechanical Engineering Korea University 145 Anam‐Ro, Seongbuk‐Gu Seoul 02841 Republic of Korea; ^2^ Department of Bionic Machinery Korea Institute of Machinery and Materials (KIMM) 156 Gajeongbuk‐ro, Yuseong‐gu Daejeon 304–343 Republic of Korea; ^3^ Department of Robotics and Mechatronics Engineering DGIST‐ETH Microrobot Research Center Daegu‐Gyeongbuk Institute of Science and Technology (DGIST) 333, Techno jungang‐daero, Hyeonpung‐Myeon Dalseong‐Gun Daegu 711–873 Republic of Korea

**Keywords:** artificial basilar membrane, cochlea, frequency discrimination, piezoelectric sensor module, spiral shape

## Abstract

A spiral‐artificial basilar membrane (S‐ABM) sensor is reported that mimics the basilar membrane (BM) of the human cochlea and can detect sound by separating it into 24 sensing channels based on the frequency band. For this, an analytical function is proposed to design the width of the BM so that the frequency bands are linearly located along the length of the BM. To fabricate the S‐ABM sensor, a spiral‐shaped polyimide film is used as a vibrating membrane, with maximum displacement at locations corresponding to specific frequency bands of sound, and attach piezoelectric sensor modules made of poly(vinylidene fluoride‐trifluoroethylene) film on top of the polyimide film to measure the vibration amplitude at each channel location. As the result, the S‐ABM sensor implements a characteristic frequency band of 96‐12,821 Hz and 24‐independent critical bands. Using real‐time signals from discriminate channels, it is demonstrated that the sensor can rapidly identify the operational noises from equipment processes as well as vehicle sounds from environmental noises on the road. The sensor can be used in a variety of applications, including speech recognition, dangerous situation recognition, hearing aids, and cochlear implants, and more.

## Graphical abstract

The spiral‐artificial basilar membrane (S‐ABM) sensor is a biomimetic acoustic sensor consisting of an artificial basilar membrane (ABM) that discriminates sound into frequency bands, 24 independent critical bands, and piezoelectric sensor modules that generates an electrical signal by the vibration of the S‐ABM. An early detection system is developed that can detect and distinguish potential hazards that may occur in industrial processes and driving situations through the sensor.

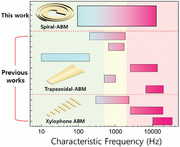

## 1 Introduction

The auditory organs of living things are biological acoustic sensors that can detect sounds in the environment and recognize them through signal processing. Among all living things, the human auditory system can detect sounds with a frequency range from 20 to 20,000 Hz and sound pressure levels ranging from 0 to 150 dB.^[^ ^1^, ^2^, ^3^, ^4^, ^5^ ^]^ The biological cochlea can discriminate the frequency of sound through the basilar membrane (BM), and using the resonance of the BM, it can recognize sounds with higher sensitivity. Therefore, acoustic sensors that mimic the biological system can present a new paradigm to the acoustic sensor market, which has been technically stagnant.

The artificial basilar membrane (ABM) sensors that discriminate frequencies according to their positions by mimicking the biological cochlea have been studied since 2005.^[^ ^6^ ^]^ In the previous studies on ABM sensors, the BM of the spiral‐shaped cochlea was modified as an unfolded, straight trapezoidal shape. Accordingly, they used to fabricate ABM as a xylophone type with a beam array^[^ ^7^, ^8^, ^9^ ^]^ or a 2D trapezoidal type with a uniform thickness.^[^ ^10^, ^11^, ^12^, ^13^, ^14^, ^15^ ^]^ However, these studies showed a narrow frequency range (less than 1 decade) as well as resonance frequency discrimination according to their channels. In particular, the straight ABMs are so lengthy and do not allow frequency discrimination in low‐frequency bands. This is because the simplified design, such as 2D modeling of 3D structures, prevented ABM from properly reflecting the frequency characteristics of biological BM. Recently, in order to solve this problem, xylophone‐type sensor research has been conducted to improve separation ability and continuity of sound frequency by increasing the number of beam arrays to 168 channels.^[^ ^16^ ^]^ Using machine learning techniques, insufficient frequency separation was compensated.^[^ ^17^ ^]^

In this study, we developed the compact, spiral‐shaped, frequency‐separated acoustic sensors that resembles biological cochlea structure. For this, we suggested a modified design scheme for the ABM sensor to mimic the structure and functional characteristics of the auditory organ, and fabricated an spiral‐artificial basilar membrane (S‐ABM) sensor with a wide frequency band similar to the human audible frequency (96‐12,821 Hz, 2.13 decades). Furthermore, the proposed S‐ABM mimics the 24 critical bands of a biological BM, decomposes sound linearly into each frequency band, and converts the vibration of the channels into electrical signals through piezoelectric materials to selectively detect the frequency of sound.

## 2 Results and Discussion

### 2.1 Biological Process Related to Frequency Discrimination of the Basilar Membrane (BM)

**Figure 1:**
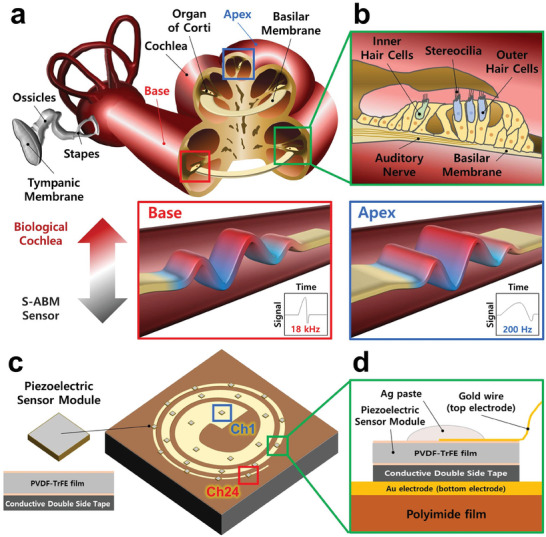
Comparison of the human cochlea and the S‐ABM sensor. a) The human's cochlea. b) The cross‐section of the organ of Corti supporting several kinds of cells. c) The S‐ABM sensor includes the suspended membrane (yellow), and piezoelectric sensors modules with square shape (grey). d) A cross‐section of the S‐ABM. Comparison of the BM vibration at the apex (blue box) and base (red box), respectively. The conceptual time‐signal graphs are in the blue and red boxes.

**Figure** **1**a,b are schematics showing cross‐sections of the human cochlea and the organ of Corti, respectively. The human ear converts sound into mechanical vibrations and perceives it in the form of electrical signals in the cochlea. The organ of the Corti has several parts that perform functions necessary for the brain to perceive sound. Among these parts, the BM is a membrane that extends from the basal end to the apical end. The BM is known to have a 3D ladder shape with a width of 100–500 µm, a thickness of 1.16–0.55 µm, and a length of 35 mm depending on the location.^[^ ^18^, ^19^, ^20^ ^]^ This structure forms different stiffness depending on the location of the BM, and the maximum vibration displacements at different locations of BM depending on the sound frequency due to differences in stiffness. The frequency of maximum vibration displacement at each position of the BM formed in this way is called the characteristic frequency. Figure 1c,d is schematics depicting an S‐ABM sensor and its cross‐section. The red and blue boxes drawn in the middle position of Figure 1 shows the BM vibrating at the base and apex positions and the conceptual time‐signal graphs, respectively. The apex is wider and thinner, so the stiffness is small, forming the maximum vibration displacement in the low‐frequency band of sounds. Conversely, the base is narrower and thicker, generating the maximum vibration displacement in the high‐frequency band of sounds. Each channel of the S‐ABM sensor is located at regular intervals, and the channel number increases from the apical end (channel 1) to the basal end (channel 24). The BM is a kind of a mechanical Fourier transform device that can discriminate signals into a series of frequencies based on its location. The decomposed frequency of sound is transmitted via the auditory nerve to the brain cortex, where it can be detected independently.^[^ ^21^, ^22^ ^]^ In 1940, Harvey Fletcher proposed the critical band, which states that human hearing perceives sound in 24 independent frequency bands. Based on this, the BM can be composed of 24 contiguous areas ≈ 1.3 mm long. This region is called critical band (CB), and it form a frequency band centered on the characteristic frequency. These critical bands allow the BM to have position‐dependent frequency selectivity.^[^ ^23^, ^24^, ^25^ ^]^

### 2.2 Design of Spiral‐Artificial Basilar Membrane (S‐ABM) Sensor

**Figure 2:**
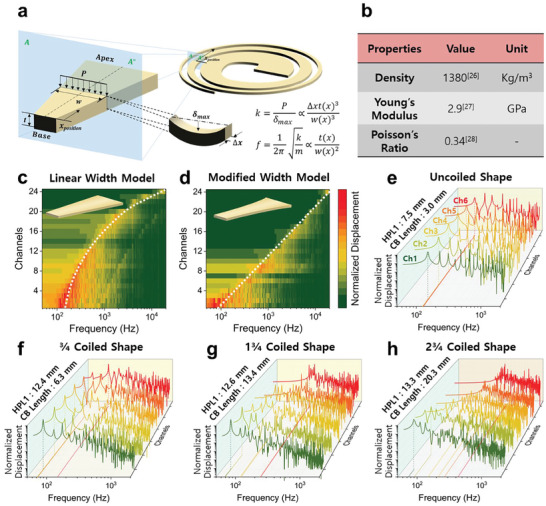
Two theoretical concepts for S‐ABM sensors that mimic the auditory perception processes and functions. a) The approximated S‐ABM as a straight‐shaped trapezoid and approximated the locally divided trapezoids as an array of beams. b) The mechanical properties of the polyimide (PI) film used as the S‐ABM. c) to h) Heatmap images of the frequency response characteristics of the S‐ABM sensor. c)The linear width model. d) The modified width model. e) The frequency discrimination capability of the uncoiled ABM. f) the coiled in ^3^/_4_ turns. g) The coiled in 1^3^/_4_ turns. h) The coiled in 2^3^/_4_ turns.

The biological BM is a 3D structure with a thickness that varies depending on its location. The apex's wider and thinner design creates lower stiffness, allowing for greater displacement at low frequencies. In contrast, the narrower and thicker base optimizes high‐frequency vibration due to its increased stiffness. The BM can be adjusted to the frequency band depending on the designed shape. **Figure** **2**a depicts the BM assumed to be an array of rectangular beams divided at a constant distance. A beam fixed at both ends can be found to have structural dynamic deflections such as Equation (1).

(1) $$\delta_{\max}=\frac{P_{\mathrm{sound}}\Delta xw^{4}}{384\mathrm{EI}}\;$$

where, δ_*max*_ is the maximum deflection of a rectangular beam, *P_sound_* is the sound pressure, Δ*x* is the x‐increment, *w* is the width of the BM, *E* is the Young's modulus of the BM and *I* is the area moment of inertia.

Using this deflection formula, a characteristic frequency calculation formula such as Equation (2) can be derived.

(2) $$f_{\mathrm{CF}}=\frac{1}{2\pi }\;\;\sqrt{\frac{k_{\mathrm{BM}}}{m_{\mathrm{BM}}}}=\frac{1}{2\pi }\;\sqrt{\frac{32E}{\rho_{\mathrm{BM}}}}\frac{t}{w^{2}}$$

where, *k_BM_* is the stiffness, *m_BM_* is the mass, *f_CF_* is the characteristic frequency and ρ_*BM*_ is the density and *t* is the thickness of the BM.

In general, the biological BM is a 3D shape with linearly varying width and thickness from the basal to the apical end. This shape makes the characteristic frequency of the biological BM to increase exponentially by moving toward the apex. In the case of an ABM, it is very difficult to manufacture a 3D shape with a thickness that varies depending on the location. Therefore, a commercial film with a uniform thickness is generally used. In the case of an ABM with a uniform thickness, the characteristic frequency increases linearly with position. In this study, to implement the characteristic frequency characteristics of the biological BM, Equation (3) was derived by reflecting the change in stiffness due to the thickness change of the ABM to the width change.

(3) $$w\left(x\right)\;=\sqrt[{4}]{\frac{32E}{\rho_{\mathrm{BM}}}}\;\sqrt{\frac{t}{2\pi f_{L}}}10^{\left\{\frac{1}{2L}\log\left(\frac{f_{L}}{f_{H}}\right)\right\}x}$$

where, *w*(*x*) is the width of the BM along the x‐axis, *f_L_* is the lowest frequency in the apical end of the BM, *f_H_* is the highest frequency in the basal end of the BM and *L* is the length of the BM.

Equation (3) is a shape in which the width of the ABM increases exponentially with position. The size of the model can be designed according to the target frequency band of the sensor. Simulations were performed using COMSOL Multiphysics, and Figure 2b shows the mechanical properties of the polyimide (PI) film used as the BM. Figure 2c,d is the results of analyzing the frequency response characteristics of a linear width model of S‐ABM that does not consider the thickness change of the BM and a modified width model of S‐ABM in which the width changes in a curved shape considering the thickness change, respectively. The x‐axis of the heatmap image represents the frequency expressed in log scale, the y‐axis is the channel divided at a constant interval, and the color of the image represents the normalized displacement of the vibration. As a result of the calculation of the linear width model, the distribution of characteristic frequencies calculated from the center of each of the 24 channels of the S‐ABM sensor shows a non‐linear tendency as the number of channels increases. On the other hand, as a result of the modified width model, the distribution of characteristic frequency calculated at the center of each channel of the S‐ABM sensor shows a linear trend as the channel increases. This suggests that the distribution of critical bands on the biological basement membrane, spaced at regular intervals, and the characteristic frequencies corresponding to their center frequencies, align with the characteristic frequency distribution observed in the S‐ABM sensor. In addition, the modified width model also implements the same feature where the bandwidth of the critical band is not constant depending on the location of the biological BM and becomes wider toward the basal end.

The most important factor in implementing the frequency discrimination ability of the biological BM is the length of the BM. In the case of a BM with a varying stiffness at each position, the displacement and wavelength of the vibration are differently formed in the critical band where the maximum displacement of the vibration occurs. Figure S1 (Supporting Information) explains the physical length of the BM and the formation of the critical band. In this study, the wavelength in the critical band is called the physical length. Figure S1a (Supporting Information) shows the 1st, 2nd, and 3rd vibration modes of a straight‐line BM calculated through simulation, and Figure S1b (Supporting Information) shows a graph showing the vibration shape of the ABM at each position from the side. When the BM is in the 1st vibration mode, the maximum vibration occurs at the apical end position with the widest width, and the longest physical length is formed. The physical length of the maximum vibration in the 2nd vibration mode and the 3rd vibration mode is gradually shortened. In theory, if the length of the critical band of the ABM is longer than half the physical length of the longest physical length, all the characteristic frequencies can be discriminated at each position. Figure 2e–h is the results of analyzing the frequency response characteristics of the BM that was coiled in a spiral shape within a limited area of 9 × 9 cm^2^, depending on the number of turns. In the case of a straight‐line ABM that is not coiled in, the HPL_1_ in the 1st vibration mode is 3.0 mm, while the length of the critical band is 7.5 mm, which is smaller. As a result, the frequency discrimination does not occur in the low‐frequency band (channels 1 to 8 among 24 channels) of the straight‐line ABM with a short length. In the case of an S‐ABM that is that was coiled in, the HPL_1_ does not change much as the number of turns increases, but the length of the BM and the length of the critical band become much longer. As a result, frequency discrimination occurs in the low‐frequency band as the number of turns increases, and all low‐frequency bands of the S‐ABM that was coiled in 2^3^/_4_ turns, like the biological BM, form different characteristic frequencies and independent critical bands.

### 2.3 Feasibility Study of S‐ABM Sensor

**Figure 3:**
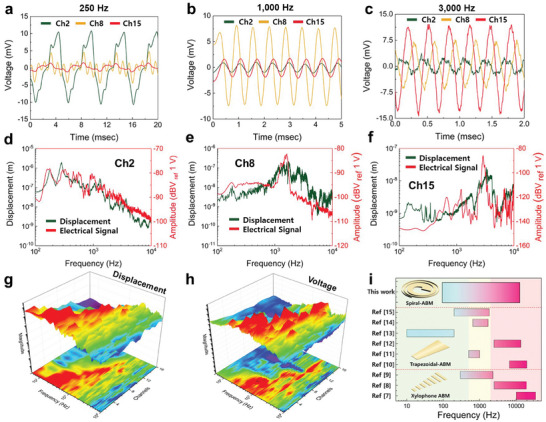
a–c) The electrical signals measured from channels 2, 8, and 15 when a single frequency signal of 250, 1,000, and 3,000 Hz was applied. d–f) The frequency response characteristics of the displacements and electrical signals measured from channels 2, 8, and 15. g) and h) The displacement and electrical signal measured from the 1 to 16 channels of the S‐ABM sensor. i) A graph comparing the ABM sensors and their frequency ranges in this and previous studies.

**Figure** **3** reveals the feasibility of the sound‐induced vibration displacement and electrical signal of the S‐ABM sensor. The vibration displacement at each channel of the BM was measured by using a laser doppler vibrometer (LDV), and the electrical signal was measured with an oscilloscope. Figure 3a–c shows the electrical signals measured from channels 2, 8, and 15 of the S‐ABM sensor. The characteristic frequencies of channels 2, 8, and 15 of the S‐ABM produced in the experiment were 177, 1,622, and 3,315 Hz, respectively. In the experiment, single‐frequency signals of 250, 1,000, and 3,000 Hz were applied to the S‐ABM. As a result of the experiment, the electrical signal was generated significantly at the electrode channel corresponding to the characteristic frequency for each single‐frequency signal, and the signal was generated weakly at other channels. Figure 3d–f displays a comparison of the vibration displacement and the electrical signal from channels 2, 8, and 15 of the S‐ABM sensor. The vibration displacement and electrical signal of channel 2 have a maximum amplitude at 272 and 177 Hz, respectively. The vibration displacement and electrical signal of the channel 8 produce a maximum amplitude at 1,731 and 1,622 Hz, respectively. Finally, the vibration displacement and electrical signal of channel 15 show a maximum amplitude at 3,315 and 2,893 Hz, respectively. It proves that the maximum vibration displacement and electrical signal of the S‐ABM are located at the same frequency, forming a characteristic frequency. Additionally, the maximum vibration displacement and electrical signal of S‐ABM do not match. It is expected that the frequency of the maximum electric signal has changed due to the influence of the mass and stiffness of components such as the piezoelectric sensor module and electrodes present on the S‐ABM. This difference in characteristic frequency can be resolved by manufacturing the piezoelectric sensor module and electrodes to a very thin thickness through a deposition process. Figure 3g,h shows the frequency response characteristics of the vibration displacement and piezoelectric voltage measured from each channel of the S‐ABM sensor. A sinusoidal sweep signal was used as sound to excite the S‐ABM sensor, increasing the frequency from the 50 Hz to 20,000 Hz in a logarithmic scale. As a result of measuring the vibration displacement using LDV, the characteristic frequencies of channels 1 and 24 of the S‐ABM sensor were confirmed to be 96 and 12,821 Hz, respectively, with a frequency range of 2.13 decades. However, since the frequency range that the mouse simulator and speaker used in the experiment can generate a constant output is 100–10,000 Hz, data from 16 channels out of the 24 channels measured were used. As a result of measuring the vibration displacement using LDV, the characteristic frequencies of channels 1 and 16 of the S‐ABM sensor were 96 and 9,816 Hz, respectively, forming a frequency band of ≈2.01 decades. In addition, it can be confirmed that the position of the maximum vibration displacement and electrical signal of the S‐ABM moves from the apical end to the basal end as the sound frequency increases. This shows that the S‐ABM discriminates the vibration of sound from each channel according to frequency and can also detect the discriminated electrical signal. It can be seen that the channel where the maximum vibration displacement due to resonance occurs at a specific frequency continues to be detected as the frequency increases. This is due to the vibration displacement caused by the 2nd and 3rd resonance frequencies after exceeding the 1st resonance frequency. As the S‐ABM progresses to the basal end, the width narrows and the stiffness increases, so the vibration displacement size of the vibration occurring at each location becomes smaller. The vibration displacement and electrical signal are largest at channel 1, which has the widest width, and the vibration displacement and voltage become smaller as they go to channel 16. In conclusion, the results of Figure 3 shows that the S‐ABM has frequency selectivity and can detect the discriminated electrical signal through the electrode channel, which can detect the frequency of sound. Figure 1d is a graph comparing the frequency ranges of ABM sensors for previous and current studies. ABM sensors would utilize the resonance of the BM, making it difficult to form a wide frequency range due to structural limitations. It can be seen that they are divided into three frequency ranges: below 500, 500–2,000, and above 2,000 Hz. In order to detect sound without frequency‐dependent sensitivity differences and distortion, it is necessary to cover all frequency ranges. Our S‐ABM sensor can measure all three frequency ranges, so it can detect and analyze sound without distortion.

### 2.4 Validation of Two Concepts for S‐ABM Design

**Figure 4:**
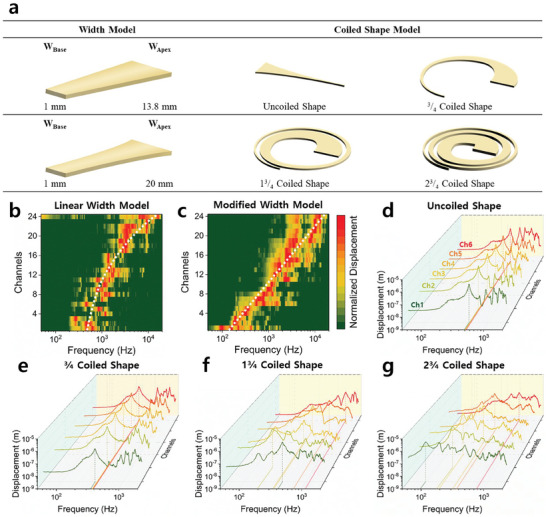
Experiments to the two theoretical concepts for S‐ABMs are simulated in Figure 2. a) The images of S‐ABM fabricated for experimental verification of the width model and the coiled shape model. b) and c) The heatmap images of the frequency response characteristics of the vibration displacement of the width model. d) the frequency response characteristics of the vibration displacement of the coiled shape model. e) the coiled in ^3^/_4_ turns. f) the coiled in 1^3^/_4_ turns. g) the coiled in 2^3^/_4_ turns.

**Figure** **4** shows the frequency response characteristics of the two concepts and the S‐ABM sensor fabricated to imitate the auditory cognitive function of the biological BM. Figure 4a displays a schematic design with the two design variables of the S‐ABM. The first design variable, width model, compares two models with linear changes in the width of the S‐ABM at each position, linear width, and modified width, which changes exponentially using Equation (3) that reflects the thickness change of the biological BM by the width change. The S‐ABM sensor used in the experiment was modeled as an S‐ABM that was coiled in 2^3^/_4_ turns within a limited area of 9 × 9 cm^2^. In the case of the modified width model, in which the width of the BM changes greatly at each position, the S‐ABM can be designed within a limited area, while in the case of the linear width model, in which the width changes little depending on the position, the S‐ABM cannot be designed within a limited area. Therefore, the S‐ABM of the linear width model used in this experiment was designed to have a linear width change of 1–13.8 mm. Figure 4b,c shows the channel‐specific frequency response characteristics measured using LDV of the linear width model and the modified width model, respectively. In Figure 4b, in the case of the linear width model, in which the thickness change of the biological BM is not reflected in the width change, the characteristic frequency of the S‐ABM at each position increases nonlinearly when the x‐axis of the frequency response characteristic is expressed in log scale. This means that the length of the critical band is the same in all channels of the S‐ABM. However, in the case of the biological BM, the characteristic frequency of the S‐ABM at each position increases exponentially from the apical end to the basal end, and the length of the critical band increases in the frequency response characteristic expressed in log scale. In Figure 4c, in the case of the modified width model, in which the thickness change is reflected in the width change, the characteristic frequency and critical band of the S‐ABM are implemented in the same way as the biological BM through the frequency response characteristic. It was confirmed that the frequency response characteristics of the two models measured through experiments are the same as the frequency response characteristics predicted by the computer simulation in Figure 2a,b, and the equation proposed in this study for designing the width of the S‐ABM can perfectly implement the distribution of characteristic frequency and critical band at each position of the biological BM.

The second design variable is the number of turns of the S‐ABM. The simulation in Figure 2c–f showed that the BM must be long enough for the frequency to be discriminated and detected in the low‐frequency band channels. To increase the length of the S‐ABM within the limited area of the sensor, the number of turns must be large. The coiled shape model in Figure 4a shows 3 schematic designs of S‐ABMs that are coiled in ^3^/_4_, 1^3^/_4,_ and 2^3^/_4_ turns, respectively. In Figure 4d, all the channels in the low‐frequency band of the uncoiled linear ABM (channels 1–6) were formed with a characteristic frequency of 509 Hz. This means that the frequency was not discriminated in the 6 channels. In Figure 4e, the S‐ABM that was coiled in ^3^/_4_ turns also formed the same characteristic frequency of 378 Hz in the 6 channels, and the frequency was not split. The reason why the characteristic frequency was not properly discriminated in the low‐frequency band can be considered as follows. The critical band length of the two models was 3.0 and 6.3 mm, respectively, which were shorter than the half‐physical length of the 1st vibration mode (HPL_1_ = 7.5 and 12.4 mm, respectively), so they did not meet the minimum length for frequency discrimination. On the other hand, the S‐ABM that was coiled in 1^3^/_4_ turns has a longer BM, with a critical band length of 13.4 mm and HPL_1_ of 12.6 mm, which is slightly longer than the critical band length. In Figure 4f, channels 1, 2, and 6 of the S‐ABM that was coiled in 1^3^/_4_ turns formed the same characteristic frequency of 453 Hz, and channels 3, 4, and 5 formed characteristic frequencies of 275, 1,162, and 834 Hz, respectively. Compared to the previous two models, the frequency was relatively well discriminated. However, the length of the critical band of the S‐ABM that was coiled in 1^3^/_4_ turns was almost the same as that of HPL_1_, so there was no clear difference, and it did not form 6 independent characteristic frequencies in all channels. In addition, the characteristic frequency of channel 3 was formed lower than that of channels 1 and 2, and the characteristic frequency did not form with a trend according to the channel position. Finally, the critical band length of the S‐ABM that was coiled in 2^3^/_4_ turns was designed to be 20.3 mm, which is significantly longer than HPL_1_ of 13.3 mm. In Figure 4g, all 6 channels in the low‐frequency band formed independent characteristic frequencies. Through the experiments of the two concepts, we established the linear distribution of the critical band of the S‐ABM and the design function for it. In addition, we proved that the length of the S‐ABM is an important variable for implementing frequency discrimination, and that the minimum length condition of the critical band must be met to implement independent frequency discrimination in all channels.

### 2.5 Applications of S‐ABM Sensor

**Figure 5:**
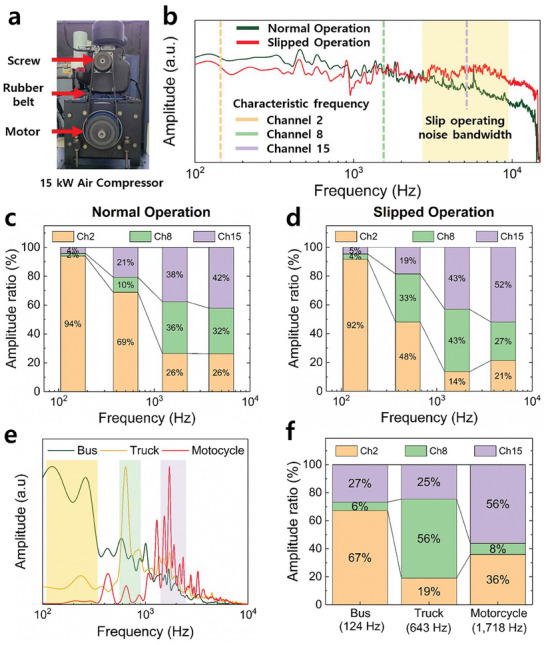
a) A photograph of a 15 kW air compressor used in a manufacturing process. b) A frequency response characteristic of the noise data generated from the operating air compressor measured by the S‐ABM. c) and d) The results of measuring the noise data during normal operation and when the slip occurs at channels 2, 8, and 15, respectively. e) A graph of the frequency response characteristics of the noise data of buses, trucks, and motorcycles. f) The results of measuring the noise data of vehicles at channels 2, 6, and 10 of the S‐ABM sensor.

We have developed a multi‐channel S‐ABM that can selectively detect frequencies. We have utilized it in areas where sound needs to be analyzed individually according to frequency. First, we applied it as a technology that can early detect problems by analyzing noise generated in work environments such as facility processes. The experiment used the noise data of a 15 kW air compressor. **Figure** **5**a shows a picture of the air compressor used in the experiment. The air compressor transmits power between the motor and the screw using a rubber belt. As the air compressor rotates, the pressure and temperature acting on the rubber belt increase, causing the rubber belt to stretch and slip. This slip phenomenon during operation causes continuous wear on the rubber belt, resulting in belt breakage. Figure 5b is a graph of the frequency response characteristics of the noise data generated during the operation of the air compressor. The black and red lines represent the noise data in the normal operating state and the noise data when slip occurs, respectively. As a result of analyzing the frequency response characteristics, the same tendency of amplitude according to frequency occurs in the low‐frequency band below 1 kHz. However, the noise generated from the slip is amplified in the 2–10 kHz frequency band and shows a difference from the normal operating state. We measured the noise data of the air compressor using a S‐ABM sensor. The noise data was measured using channels 2, 8, and 15, with respective channel characteristic frequencies of 177, 1,622, and 3,315 Hz. The critical band of channel 15 is in the anomalous frequency band where the sound difference occurs when slip occurs during the operation of the air compressor. As a result of the experiment, in the normal operating state, the ratio of the electrical signal between channel 15, which was measured in the anomalous frequency band, and other channels was 42%. However, after slip occurred, the value of the electrical signal increased, and the ratio increased to 52%. On the other hand, the electrical signal ratios of channels 2 and 8 decreased from 26% and 32% to 21% and 27%, respectively. On the road, we see various types of vehicles, from small, slow‐moving vehicles to large, very fast‐moving vehicles. These vehicles also generate unique driving sounds depending on the vehicle type. By analyzing these driving sounds, we can know the type and number of vehicles passing by on the road, and we can also early detect emergency situations such as traffic accidents. Figure 5e is a graph of the frequency response characteristics of the noise data of bus, truck, and motorcycle that can be heard on the road. The three types of vehicles form a frequency band centered on 124, 643, and 1,718 Hz, respectively. The vehicle frequency was measured using channels 2, 6, and 10, with channel characteristic frequencies of 177, 612, and 1,812 Hz, respectively. As a result of the experiment, the ratio of the electrical signal in the channel corresponding to the frequency band of each vehicle's driving sound was the largest, with 67% for the bus on channel 2, 56% for the truck on channel 6, and 56% for channel 10 for the motorcycle. We have verified that the S‐ABM sensor can detect changes in the signal value of a specific frequency band through frequency discrimination, and that this can be used to early detect various types of problem situations. The S‐ABM, which can detect independent frequency bands, has a different operating principle from conventional acoustic sensor technologies, and can play a major role in environments that are difficult to solve with conventional technologies alone.

## 3 Conclusion

We have developed a S‐ABM sensor that can mimic the human auditory system to discriminate sound according to frequency bands and detect them selectively. To mimic the frequency discrimination and perception tendencies of the biological BM, we proposed an optimized design equation with the material properties, size, and target frequency range as variables. The optimized design equation reflects the thickness change of the biological BM to the width change to mimic the change in stiffness of the BM depending on its position, as in the biological BM. It also proposes the concept of the minimum length of the BM that can form independent characteristic frequencies and critical bands in the lower frequency band. In this way, we fabricated an ABM in a spiral shape within a limited area. The length of the S‐ABM designed in this way is at least five times longer than that of the straight shape. We fabricated a S‐ABM with 2^3^/_4_ turns and attached PVDF piezoelectric sensor modules to each channel to convert the vibration displacement of the S‐ABM into an electrical signal. The frequency range of the S‐ABM sensor is 96‐12,821 Hz, 2.13 decades, and it forms independent characteristic frequencies and critical bands in 24 electrode channels. Each channel of the S‐ABM could detect an electrical signal through the piezoelectric voltage caused by vibration depending on the frequency band of the sound. However, if a speaker with a constant output in the 10000–20,000 Hz range is used instead of the speaker used in the experiment, the upper frequency will be higher, and it is expected to have a wider frequency range. Additionally, the PVDF‐TrFE film used in the current PVDF sensor module lacks piezoelectrical characteristics, making it difficult to properly detect amplitudes that become smaller toward the base position. In follow‐up research, in order to complement these aspects, a sensor module can be manufactured using nanofiber‐based piezoelectric or ceramic materials that are flexible and have high piezoelectric efficiency.^[^ ^29^, ^30^, ^31^ ^]^ The S‐ABM sensor can detect abnormal operation of equipment by analyzing noise generated in the facilities and can distinguish the type of vehicles driving along the road by frequency. The S‐ABM sensor developed in this way is expected to have great potential in fields such as speech recognition that can distinguish speakers early warning systems that can detect dangerous situations early, and high‐performance cochlear implants.

## 4 Experimental Section

### Fabrication of S‐ABM Sensor

The S‐ABM in this study was designed and optimized based on Equation (3), and the performance of the model was verified using simulations. A 12.5 µm thick polyimide (PI) film was used as the S‐ABM, and Figure 2b shows the mechanical properties of the PI film used.^[^ ^21^, ^22^, ^23^ ^]^ One of the variables required for designing the width of the BM is the target frequency band of the sensor. The S‐ABM was designed to have a frequency band of 100–20,000 Hz, which is the human audible frequency band. Frequencies below 100 Hz were excluded to avoid the influence of the power noise (60 Hz) of the measurement equipment used in the experiment. As a result, the selected width of the BM was designed to be 1–20 mm. Figure S3 (Supporting Information) shows the process of fabricating the S‐ABM sensor. A 9 × 9 cm^2^ Photopolymer body was fabricated using a 3D printer. The center of the body was pierced in the shape of the BM, and the upper surface is coated with an adhesive layer using double‐sided tape. Currently, the part that is pierced in the center of the body is removed along the outer shape of the BM using laser cutting. Next, a 12.5 µm thick PI film was attached to the surface of the body with an adhesive layer to use as the S‐ABM. The lower electrode with a width of 1 mm was deposited on the surface of the S‐ABM using an E‐beam evaporator. A polymer film was used as a mask, and gold was used as the material for deposition. A sensor module and upper electrode were fabricated in the center of the 24 critical bands, which were divided into the S‐ABM at regular intervals, to measure electrical signals. The sensor module is a square with a size of 1.5 × 1.5 mm^2^ and a thickness of 28 µm, and poly(vinylidene fluoride‐trifluoroethylene) (PVDF‐TrFE) film was used as the material. The bottom of the film was attached to an aluminum conductive tape to connect to the lower electrode. The gold wire was fixed to the top of the sensor module and connected to the PCB board using jumper wires. Figure S3a,b (Supporting Information) show the finished S‐ABM sensor and a schematic of the cross‐section, respectively. As shown in Figure 1f, the PI film plays the role of the BM that discriminates frequencies, the sensor module plays the role of the IHCs that detect the vibration of sound and convert it into an electrical signal. The gold electrode also plays the role of the auditory nerve that outputs the electrical signal detected by the vibration. The individual parts of the S‐ABM mimic the individual parts of the biological cochlea and implement the auditory cognition process and function.

### Equipment and Experimental Process

The displacement and electrical signals of the S‐ABM sensor were measured in two different experimental processes. Figure S4a (Supporting Information) shows the setup and process of the experimental equipment for measuring the displacement of the S‐ABM. The displacement was measured using a LDV. The LDV used in the experiment was the PSV‐400 Scanning Vibrometer from Polytec Co. To reproduce the sound signal, the Mouth Simulator Types 4227 from B&K Co. was used. The frequency response characteristics of the product are 100–10,000 Hz, ±6 dB. The voltage of the sound source applied to the speaker was amplified using a power amplifier. The power amplifier used was the CS 3000 from Peavey Co. The frequency response characteristic is 10–100 kHz, and the voltage gain is 32 dB. The measurement process was controlled by a junction box using a PC. The junction box transmitted the sound signal to the mouth simulator, and the sound source was reproduced through the mouth simulator. The displacement of the S‐ABM was measured using an LDV. Figure S4b (Supporting Information) shows the setup and process of the experimental equipment for measuring the electrical signal of the S‐ABM. The electrical signal was measured using an oscilloscope. The oscilloscope used in the experiment was the MDO4024C from Tektronix Co. To reproduce the sound signal, the Razer Nommo Chroma from Razer Co. was used. The frequency response characteristics of the product are 50‐10,000 Hz, ±10 dB. The voltage of the sound signal applied to the speaker was amplified using an audio amplifier. The audio amplifier used was the MA400 from Behringer Co. The frequency response characteristic was 15–25 kHz, and the voltage gain was 40 dB. The measurement process was controlled by a PC. The sound signal was transmitted to the speaker through the PC, and the sound source was reproduced through the speaker. The piezoelectric voltage signal generated by the vibration of the BM was measured using an oscilloscope.

### Impact on Vibration When a PVDF Module is Added to the S‐ABM Sensor

In Figure S5a (Supporting Information), three S‐ABM sensors were fabricated: a step with only PI film, a step with Au electrodes deposited on PI, and finally a step with a PVDF module on top of the Au electrodes, and compared the frequency response characteristics. To check the effect of the materials added at each step on the characteristic frequency and amplitude, experiments were conducted at channel 1 corresponding to each apex, and channel 20 corresponding to the base. The sound source used in the experiment used a sinusoidal sweep signal, and a frequency of 50 to 10,000 Hz was applied. Figure S5b (Supporting Information) shows the frequency response characteristics in the 50 to 1,000 Hz band measured at channel 1 of the three S‐ABM sensors. There was a difference in the characteristic frequency of the sensor with only the PI film and the sensor with the Au electrode deposited, but this could be considered an experimental error. On the other hand, the characteristic frequency of the sensor with the PVDF module attached was 200 Hz, which was lower than that of the sensor with only the PI film (300 Hz). According to Equation (2), the characteristic frequency is inversely proportional to mass. It can be seen that as the PVDF module was added to the sensor with only the PI film, the mass of the S‐ABM increases and the characteristic frequency decreased. Figure S5c (Supporting Information) shows the frequency response characteristics in the 1000–10,000 Hz band measured at channel 20 of the three S‐ABM sensors. The magnitude of the displacement at channel 20 of the sensor with only the PI film was ≈ 60 nm. In the case of a sensor with a PVDF module, the displacement is ≈10 nm. This means that the damping of PVDF has been added to the sensor, and it could be predicted that the displacement of the sensor is attenuated due to the damping effect.

## Conflict of Interest

The authors declare no conflict of interest.

## Author Contributions

E.‐S.J. and C.‐S.H. conceived the project and designed the experiments. E.‐S.J. and U.L. performed all experiments and analysis. S.Y., S.H., and H.C. helped to evaluate the performance of the sensors. E.‐S.J. and C.‐S.H. wrote the manuscript.

## Supporting information

Supporting Information

## Data Availability

Data sharing is not applicable to this article as no new data were created or analyzed in this study.
