# Supplementary material for: Frequency‐Selective, Multi‐Channel, Self‐Powered Artificial Basilar Membrane Sensor with a Spiral Shape and 24 Critical Bands Inspired by the Human Cochlea
Source: Adv Sci (Weinh). 2024 Jun 17;11(31):2400955. doi: 10.1002/advs.202400955 (PMC11336941; doi:10.1002/advs.202400955)
Supplement: Supplementary file 1 — Supporting Information [file ADVS-11-2400955-s001.docx]

Supporting Information

**Frequency-selective, multi-channel, self-powered artificial basilar membrane sensor with a spiral shape and 24 critical bands inspired by the human cochlea**

Eun-Seok Jeon,^1^ Useung Lee,^1^ Seongho Yoon,^1^ Shin Hur,^2^ Hongsoo Choi,^3^ Chang-Soo Han ^1, *^

^1^ Department of Mechanical Engineering, Korea University, 145 Anam-Ro, Seongbuk-Gu, Seoul 02841,

Republic of Korea

^2^ Department of Bionic Machinery, Korea Institute of Machinery and Materials (KIMM), 156 Gajeongbuk-ro, Yuseong-gu, Daejeon 304-343, Republic of Korea

^3^ Department of Robotics Engineering, DGIST-ETH Microrobot Research Center, Daegu Gyeongbuk Institute of Science and Technology (DGIST), 333, Techno jungang-daero, Hyeonpung-Myeon, Dalseong-Gun, Daegu 711-873, Republic of Korea


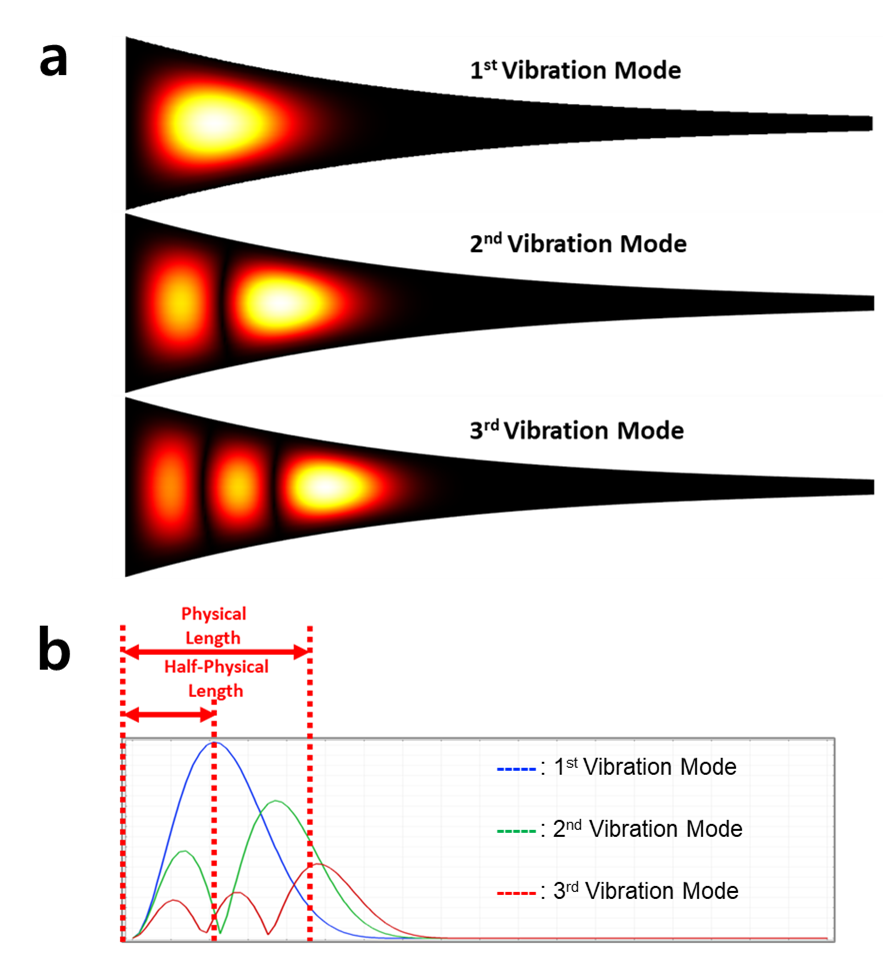


Figure S1. The relation between the length of an ABM and its frequency discrimination capability. A) The 1st, 2nd, and 3rd vibration modes of an ABM calculated through simulation, as well as the location of the maximum vibration displacement. B) A comparison the relative size of the vibration displacement at different locations of the BM.


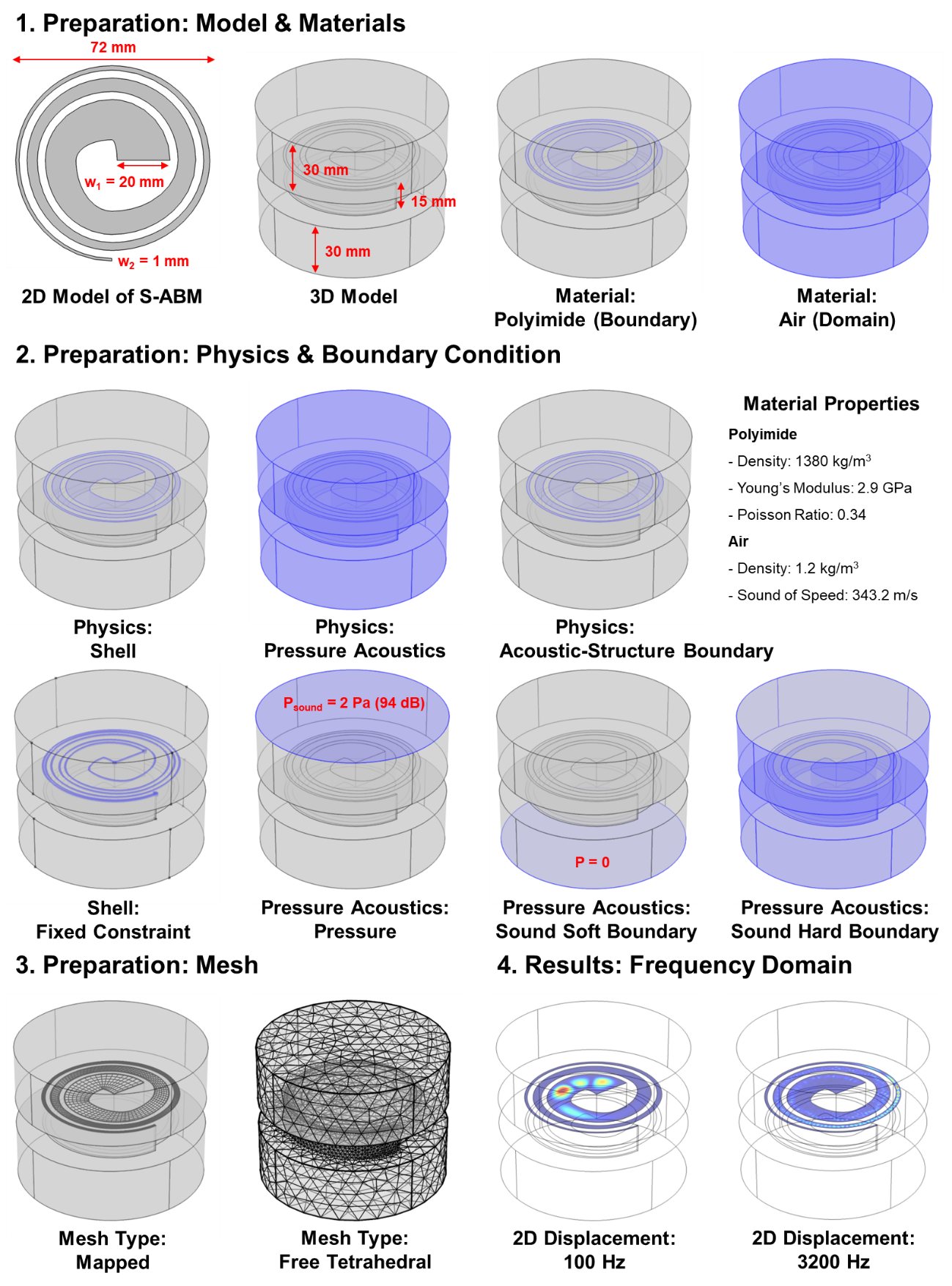


Figure S2. The simulation process in the COMSOL Multiphysics for calculating the frequency response at the S-ABM sensor. Frequency response calculation involving a four-steps process: 1) Modeling the structures and setting the material properties. 2) Choosing the physics modules and applying the boundary conditions. 3) Generating the mesh. 4) Solving the simulation and obtaining the results.


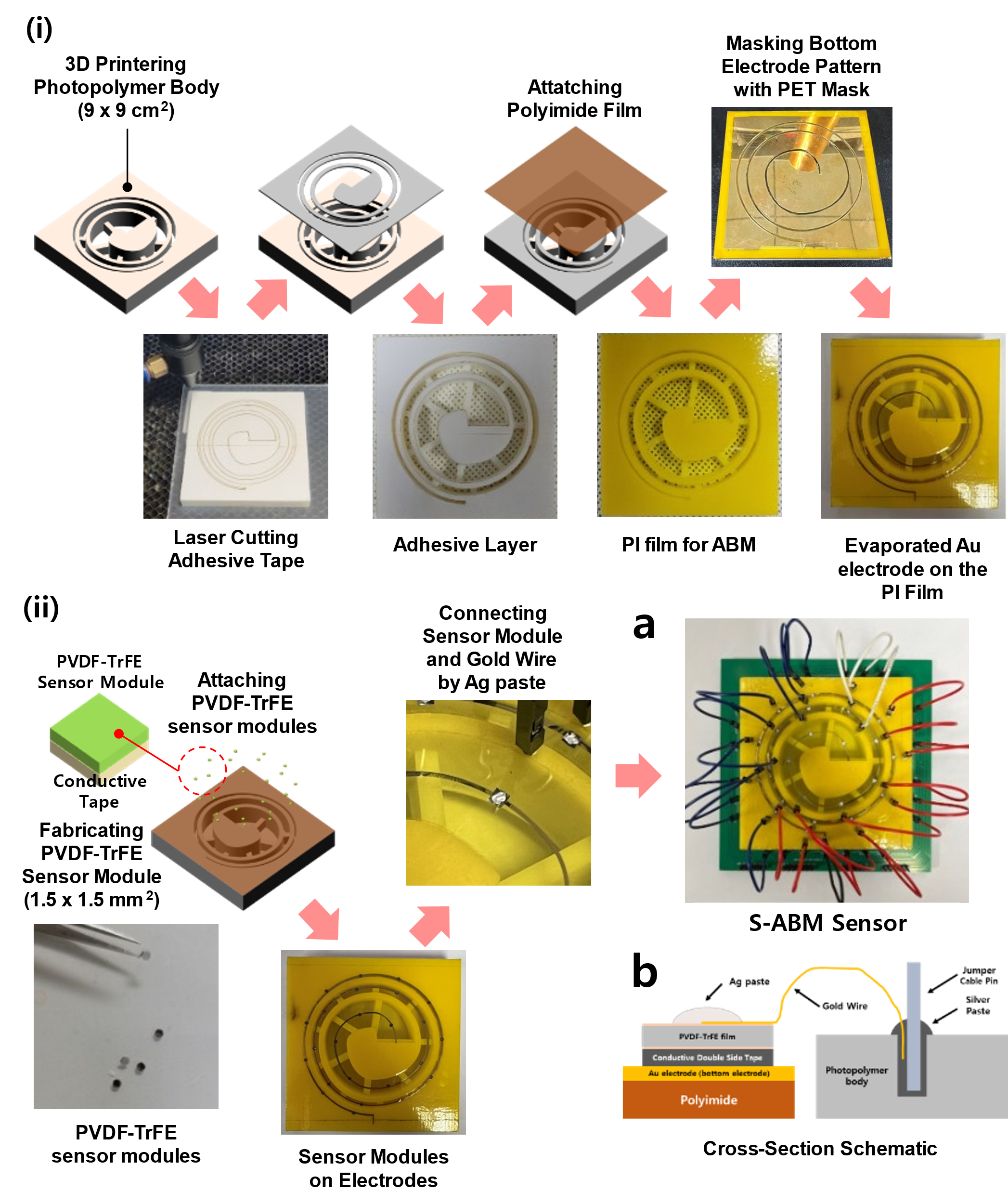


Figure S3. The fabrication process of a S-ABM sensor. A photopolymer using a sensor body fabricated by 3D printer. The double-sided tape using an adhesive layer attached on the upper surface. A PI film using a S-ABM attached on the adhesive layer. A gold layer using a bottom electrode deposited by e-beam evaporator using a along the length direction. The sensor module consisting of a poly(vinylidene fluoride-trifluoroethylene) (PVDF-TrFE) film and aluminum conductive tape. It’s attached to the center of the 24 critical bands of the S-ABM. A gold wire using a top electrode fixed on the sensor module by Ag paste and connecting with a jumper wire to the PCB board. A) An image of the S-ABM sensor. B) The cross-section of the S-ABM sensor.


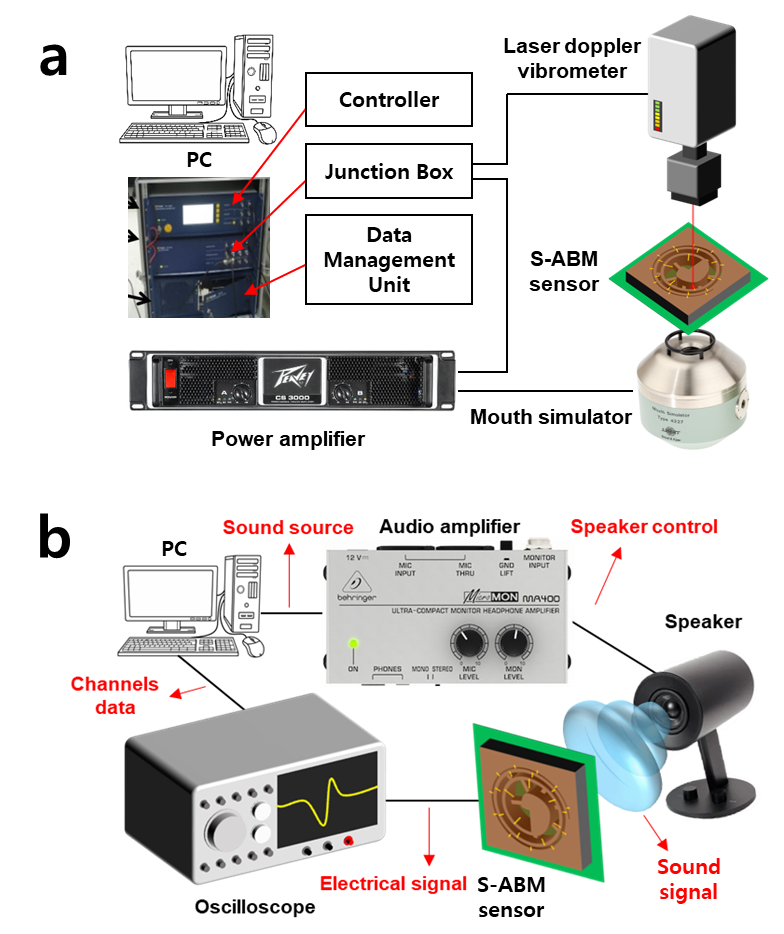


Figure S4. A) The schematic of measuring vibration displacement of the S-ABM sensor. B) The schematic of measuring electrical signal of the S-ABM sensor.

**
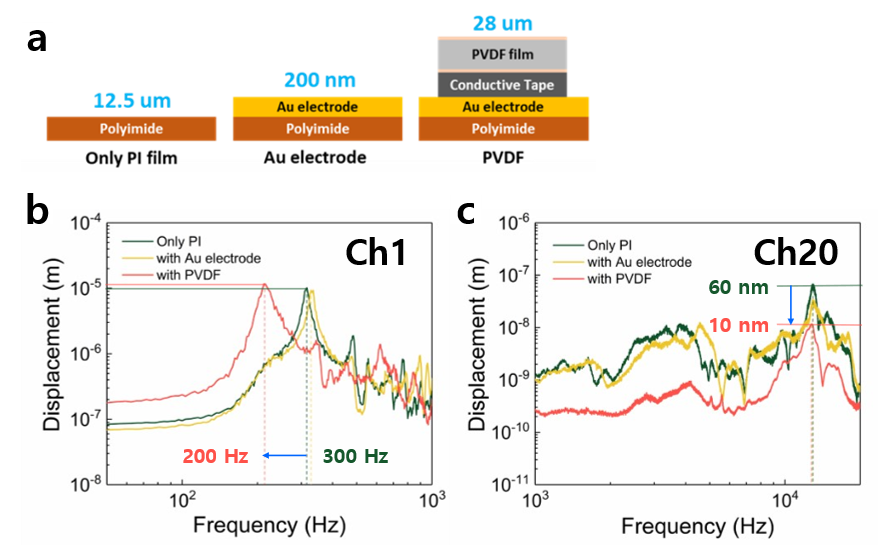
**

Figure S5. A) The schematics of three S-ABM sensors: only PI film, Au electrodes deposited on PI, and a PVDF module on top of the Au electrodes. The frequency response analysis of three S-ABM sensors in B) channel 1 and C) channel 20.
